# Efficacy and safety of single-dose intravitreal dexamethasone implant in non-infectious uveitic macular edema: A systematic review and meta-analysis

**DOI:** 10.3389/fmed.2023.1126724

**Published:** 2023-02-17

**Authors:** Shipei Fan, Xing-yu Shi, Chao-fu Zhao, Zhen Chen, Jia Ying, Song-ping Yu, Jun Li, Xia Li

**Affiliations:** ^1^Department of Ophthalmology, Lishui Municipal Central Hospital, The Fifth Affiliated Hospital of Wenzhou Medical University, Lishui, Zhejiang, China; ^2^Department of Nephrology, Lishui Municipal Central Hospital, The Fifth Affiliated Hospital of Wenzhou Medical University, Lishui, Zhejiang, China

**Keywords:** macular edema, intravitreal implant, dexamethasone, meta-analysis, non-infectious uveitis

## Abstract

**Purpose:**

We conducted a systematic review and meta-analysis to investigate the efficacy and safety of single-dose intravitreal dexamethasone (DEX) implant for treating non-infectious uveitic macular edema (UME).

**Methods:**

Studies including clinical outcomes of the DEX implant in UME were comprehensively searched in PubMed, Embase, and Cochrane databases for potential studies from inception to July 2022. The primary outcomes were best corrected visual acuity (BCVA) and central macular thickness (CMT) during the follow-up period. Stata 12.0 was used to perform the statistical analyses.

**Results:**

Six retrospective studies and one prospective investigation involving 201 eyes were ultimately included. Significantly improved BCVA was observed from baseline to 1 month (WMD = −0.15, 95%CI = −0.24, −0.06), 3 months (WMD = −0.22, 95%CI = −0.29, −0.15), and 6 months (WMD = −0.24, 95%CI = −0.35, −0.13), after single-dose DEX implant. When considering CMT, macular thickness of 1 month (WMD = −179.77, 95%CI = −223.45, −136.09), 3 months (WMD = −179.13, 95%CI = −232.63, −125.63), and 6 months (WMD = −140.25, 95%CI = −227.61, −52.88) decreased in comparison with baseline, with statistical significance.

**Conclusion:**

Based on the current results, this meta-analysis confirmed favorable visual prognosis and anatomical improvement in patients with UME, after receiving the single-dose DEX implant. The most common adverse event is increased intraocular pressure, which could be controlled with topical medications.

**Systematic Review Registration:**https://www.crd.york.ac.uk/PROSPERO/, identifier CRD42022325969.

## Introduction

Uveitis accounts for 10–15% of blindness in developed countries, with an estimated prevalence of 9–730 cases per 100,000 population (1, 2). Uveitic macular edema (UME) is the most frequent clinical complication of non-infectious uveitis and could persist for a long period despite various treatment modalities and adequate control of ocular inflammation, leading to structural retinal damage and irreversible vision impairment (3).

Large publications have focused on various aspects of UME, yet the detailed and comprehensive pathogenesis remains not fully understood. Prior investigations have found increased pro-inflammatory cytokine levels such as interleukin-6 (IL-6), IL-8, tumor necrosis factor-α, and vascular endothelial growth factor (VEGF), which might play an essential role in UME (4, 5). A recent study demonstrates that the regulatory T cell is positively associated with persistent anatomical improvement and might be a prognostic factor for patients with UME (5). In short, breakdown of the outer and inner blood-retinal barrier results in increased permeability of the microvasculature and pigment epithelium, leading to fluid accumulation and macular edema. Therefore, exploring effective and acceptable therapeutic strategies is a persistent challenge.

Local and systemic uses of corticosteroids are the first-line treatment option for UME, while long-term use may be burdened by multiple side effects, including poor blood glucose control, osteoporosis, cataract progression, and ocular hypertension (6). Therefore, different interventions including immunomodulatory agents, anti-VEGF, and pars plana vitrectomy are also adopted for UME (7). However, the prognosis remains unsatisfactory in patients with chronic and refractory UME. Thus, there has been a growing body of studies that focus on intravitreal implants to improve visual outcomes and minimize ocular side effects in recent years.

The intravitreal dexamethasone (DEX) implant (Ozurdex; Allergan, Inc., Irvine, CA) is a sustained-release implant designed to deliver 0.7 mg of dexamethasone in vitreous (8). A recently published network meta-analysis manifested that the DEX implant could improve the anatomical structure and vitreous haze of non-infectious uveitis (9). In addition, the HURON study had demonstrated significantly reduced central macular thickness (CMT) and improved visual acuity with duration for 6 months in non-infectious uveitis after a single DEX implantation (10). Although multiple publications have manifested the efficacy of the DEX implant in UME, the precise conclusion remains unclear. To elucidate the potential benefits and drawbacks of this treatment option, this meta-analysis was performed to systematically determine the effectiveness and safety of a single-dose DEX implant for macular edema secondary to non-infectious uveitis.

## Methods

The present meta-analysis was conducted based on the principles proposed by the Cochrane Handbook (11) and the Preferred Reporting Items for Systematic reviews and Meta-Analyses (PRISMA) statement (12). No ethical approval and informed consent were required. This analysis has already been registered in Prospero.

### Search strategy

In total, three electronic databases, including PubMed, Embase, and Cochrane library, were searched comprehensively in July 2022 by two independent investigators (FSP and SXY). The search strategy was performed in accordance with the following terms: ((“uveitic” [tiab]) OR (“uveitis” [tiab]) OR (“UME” [tiab]) OR (“Uveitides” [tiab]) OR (“panuveitis” [tiab]) OR (“iridocyclitis” [tiab]) OR (“vasculitis” [tiab]) OR (“(retinal vasculitis” [tiab]) OR (“ocular inflammation” [tiab])) AND ((“macular edema” [tiab]) OR (“(macular oedema” [tiab])) AND ((“intravitreal dexamethasone implant” [tiab]) OR (“(dexamethasone” [tiab]) OR (“ozurdex” [tiab])). The references of associated publications were further screened thoroughly for additional relevant investigations.

### Inclusion and exclusion criteria

Eligible studies were required to accord with the following criteria: (1) original investigation focusing on non-infectious UME; (2) chronic macular edema refractory to previous treatments; (3) the age of patients >18 years; (4) sample size of included eyes in each study was at least 20; (5) acceptance of DEX implant with at least 3 months follow-up period; and (6) the main outcomes were expressed as mean ± standard deviation (SD). The exclusion was adopted as follows: (1) patients with other fundus diseases such as diabetic retinopathy, age-related macular degeneration, and choroidal neovascularization; (2) case reports, reviews, letters, editorials, and comments without data; and (3) patients who underwent prior pars plana vitrectomy.

### Data extraction and quality assessment

Overall, two investigators (FSP and SXY) separately screened the titles and abstracts of eligible studies and assessed entire articles to evaluate the finally included investigations. The data extraction was conducted by two independent researchers (FSP and SXY) from eligible studies. From each publication, the following demographic information and clinical characteristics were extracted: first author, publication date, location and study period, study type, duration of uveitis, follow-up time, mean number of DEX implants, previous systemic and local treatments, incidence of adverse events, best corrected visual acuity (BCVA), and CMT change after single-dose DEX implant. The criteria reported by the Methodological Index for Non-randomized Studies (Minors) were adopted to evaluate the evidence quality of included studies, which contained eight items specifically for non-comparative studies (13). A third reviewer (LX) was involved in case of any disagreement to reach a consensus.

### Quantitative analysis

Stata 12.0 (Stata Corporation, College Station, TX, United States) was applied to conduct all data analyses. The inverse-variance model was utilized to determine the weight mean difference (WMD) with a 95% confidence interval (CI) for continuous outcomes in the present meta-analysis. The evaluation of statistical heterogeneity was assessed using Cochran’s *Q* test and *I*^2^ test. Based on the meta-analysis principle, a value of *I*^2^ < 50% indicated relatively low heterogeneity across studies, while *I*^2^ > 75% represented substantial heterogeneity. When significant heterogeneity was determined, a random-effect model was used; otherwise, a fixed-effect model was applied. Publication bias was calculated by the Egger test. A two-sided *p* value <0.05 was adopted as statistically significant.

## Results

### Selection of studies

**Figure 1:**
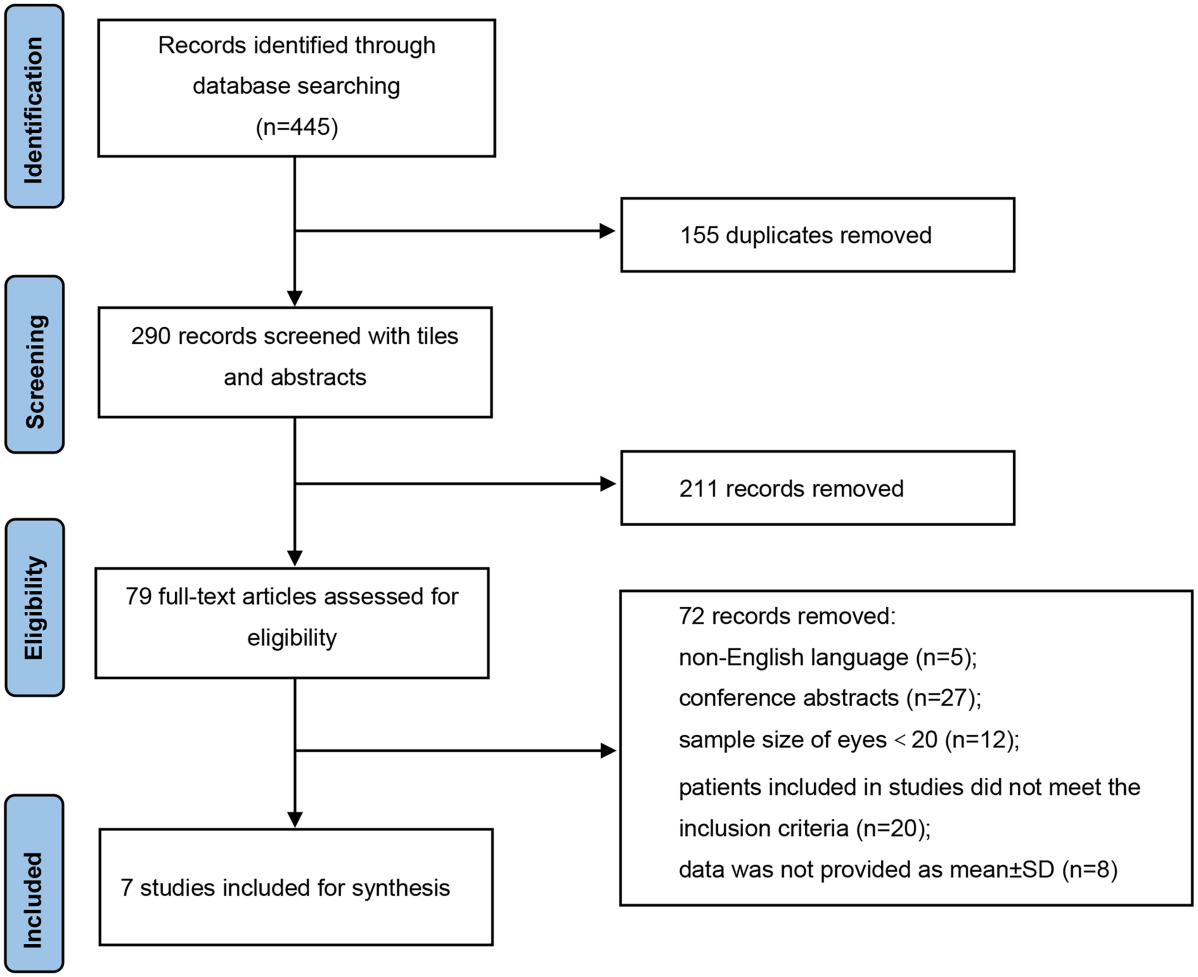
Flow diagram of the study selection process.

As illustrated in Figure 1, a total of 445 records were identified in accordance with the search strategy from electronic databases (PubMed = 265, Embase = 180), of which 155 duplicates were excluded. After screening the titles and abstracts of the remaining 290 publications, 211 studies were ruled out. Among the 79 potentially eligible records for full-text review, 72 articles were excluded. Finally, seven studies were included and pooled together for further data synthesis (14–20). Studies were ruled out for the following reasons: non-English language, conference abstracts, sample size of included eyes was less than 20, patients included in studies did not meet the inclusion criteria, and data were not provided as mean ± SD.

### Baseline characteristics

Detailed baseline demographics and clinical information are summarized in Tables 1, 2. Among seven publications, a total of 201 eyes were retrieved in accordance with the inclusion criteria. The sample size of included studies ranged from 22 to 41 eyes. In total, six studies were designed as retrospective and one study was designed as prospective. The clinical diagnoses of included studies were summarized in Supplementary Table S1. The methodologic score of eligible studies ranged from 11 to 14, indicating relatively high quality and reliability of results from included studies (Table 3).

**Table 1:** Clinical demographics of included studies.

Study	Year	Country	Survey period	Study type	Eyes (patients)	Age (years)	Duration of uveitis (years)	Follow-up period (months)
Bansal et al. (14)	2015	India	NA	Prospective	30 (27)	46.1 ± 15.7	1.4 ± 0.6	5.6
Garweg et al. (15)	2016	Switzerland	NA	Retrospective	26 (19)	NA	NA	NA
Fabiani et al. (16)	2017	Italy	NA	Retrospective	22 (22)	49.0 ± 20.1	3.5 ± 2.5	6
Cardoso et al. (17)	2017	France	2012–2013	Retrospective	41 (31)	57.9 ± 13.1	NA	13.4 ± 5.9
Tsang et al. (18)	2017	Canada	2012–2014	Retrospective	25 (15)	46.8	NA	9
Nagpal et al. (19)	2018	India	2013–2016	Retrospective	30 (NA)	NA	NA	6
Yalcinbayir et al. (20)	2019	Turkey	2013–2016	Retrospective	27 (20)	35.6 ± 12.1	NA	24.4 ± 9.9

NA, Not applicable.

**Table 2:** Demographics of included studies.

Study	Mean number of implants	Previous systemic treatments			Prior ocular treatments		
		Steroid	Immunosuppressant	Biological agent	Anti-VEGF	IV steroid	ST steroid
Bansal et al. (14)	1.1	27 patients	8 patients	NA	11 patients	NA	NA
Garweg et al. (15)	NA	NA	NA	NA	NA	NA	NA
Fabiani et al. (16)	1	19 patients	13 patients	10 patients	NA	NA	NA
Cardoso et al. (17)	1.4	21 patients	12 patients	4 patients	NA	NA	2 patients
Tsang et al. (18)	1.4	5 patients	5 patients	1 patient	No	3 patients	11 patients
Nagpal et al. (19)	1	NA	NA	NA	2 patients	NA	NA
Yalcinbayir et al. (20)	1.2	NA	12 patients	8 patients	NA	NA	NA

VEGF, Vascular endothelial growth factor; IV, Intravitreal; ST, Sub-tenon; NA, Not applicable.

**Table 3:** The quality of included studies based on the MINORS.

Study	−1	−2	−3	−4	−5	−6	−7	−8	Total
Bansal et al. (14)	2	2	2	2	2	2	2	0	14
Garweg et al. (15)	2	2	0	2	2	1	2	0	11
Fabiani et al. (16)	2	2	0	2	2	2	2	0	12
Cardoso et al. (17)	2	2	0	2	2	2	1	0	11
Tsang et al. (18)	2	2	0	2	2	2	2	0	12
Nagpal et al. (19)	2	2	0	2	2	2	2	0	12
Yalcinbayir et al. (20)	2	2	0	2	2	2	2	0	12

Studies fulling the criteria of: (1) clearly stated aim; (2) consecutive patients; (3) prospective data collection; (4) endpoints appropriate to the aim of the study; (5) unbiased assessment of the study endpoint; (6) follow-up period appropriate to the aim of the study; (7) loss to follow up less than 5%; (8) prospective calculation of the study size. MINORS, Methodological item for non-randomized studies; 0 = not reported; 1 = reported, but inadequate; 2 = reported and adequate.

### Best corrected vision acuity and central macular thickness

**Figure 2:**
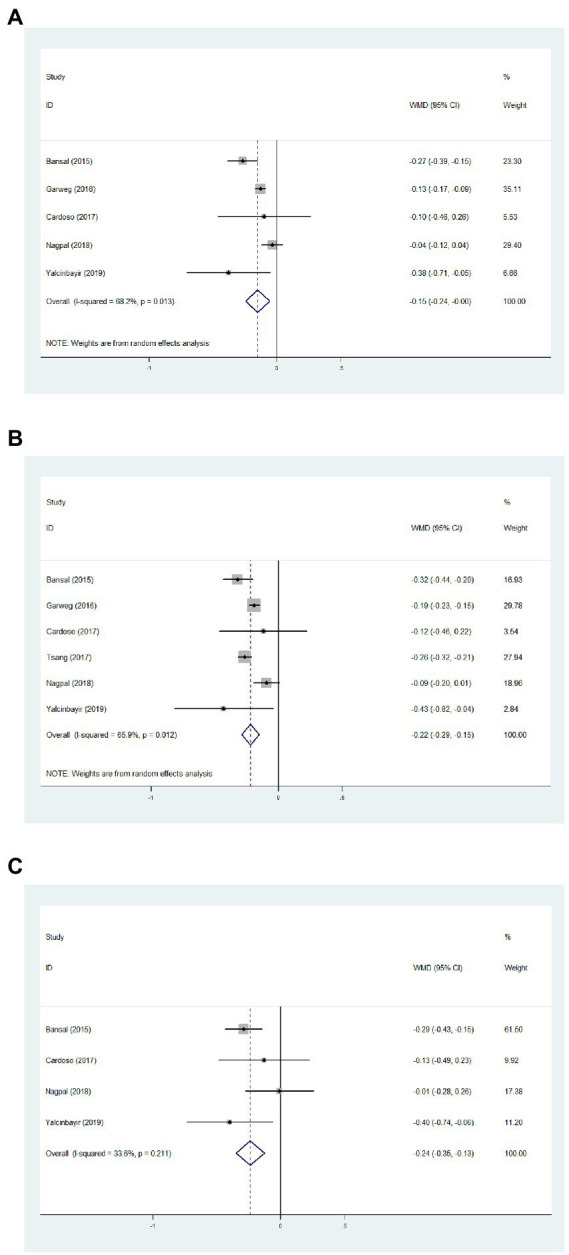
Forest plot showing best corrected visual acuity changes from baseline to 1 **(A)**, 3 **(B)**, and 6 months **(C)**.

Data from five studies, six studies, and four studies were pooled together to determine the average change of BCVA from baseline to 1, 3, and 6 months, respectively. After the DEX implant, BCVA significantly increased at 1 month, compared to the baseline (Figure 2A, WMD = −0.15, 95%CI = −0.24, −0.06, *p* = 0.013, *I*^2^ = 68.2%). At 3 months, significantly improved BCVA was determined (Figure 2B, WMD = −0.22, 95%CI = −0.29, −0.15, *p* = 0.012, *I*^2^ = 65.9%). When considering 6 months, BCVA improved from the baseline with an average of −0.24 logMAR (Figure 2C, WMD = −0.24, 95%CI = −0.35, −0.13, *p* = 0.211, *I*^2^ = 33.6%).

**Figure 3:**
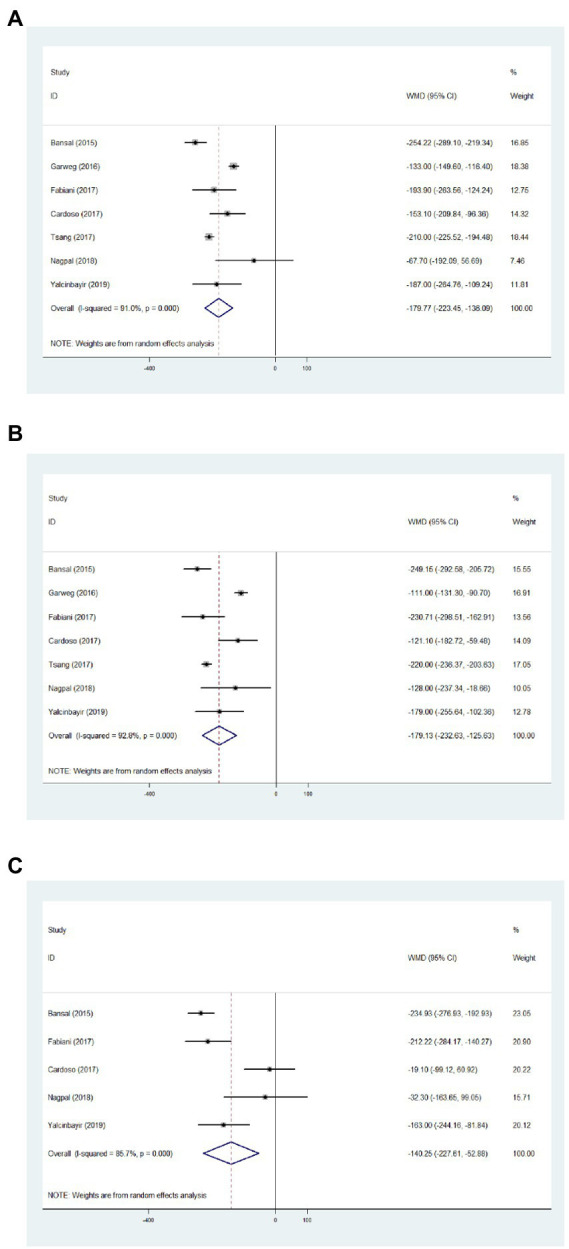
Forest plot showing central macular thickness changes from baseline to 1 **(A)**, 3 **(B)**, and 6 months **(C)**.

Regarding the CMT of 1 month, results from seven articles identified significantly reduced thickness with an average of −179.77 μm, compared with baseline data (Figure 3A, WMD = −179.77, 95%CI = −223.45, −136.09, *p* < 0.001, *I*^2^ = 91.0%). In addition, significantly decreased CMT was observed at 3 months in comparison with baseline (Figure 3B, WMD = −179.13, 95%CI = −232.63, −125.63, *p* < 0.001, *I*^2^ = 92.8%). Significant difference between the CMT of 6 months and baseline was determined (Figure 3C, WMD = −140.25, 95%CI = −227.61, −52.88, *p* < 0.001, *I*^2^ = 85.7%).

### Adverse events and publication bias

**Figure 4:**
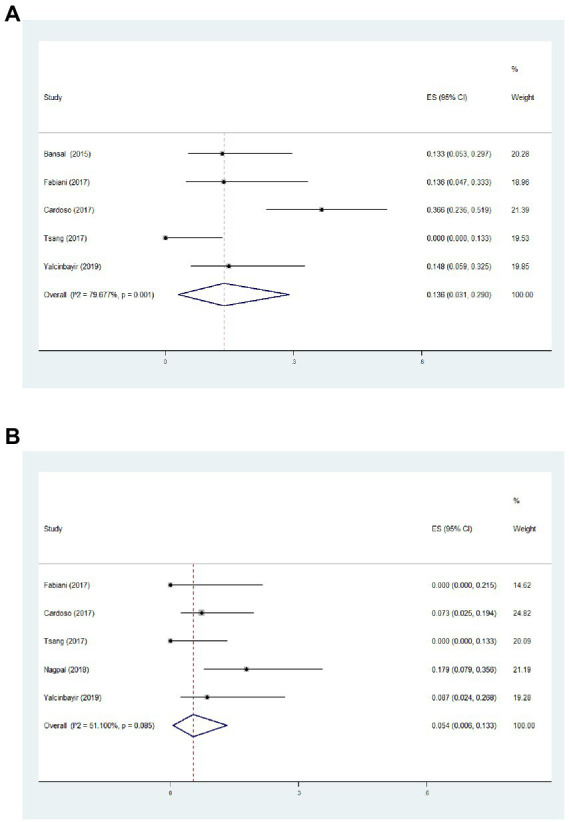
Forest plot of incidence of ocular hypertension **(A)** and cataract formation **(B)** during follow-up.

Based on this meta-analysis, it had been revealed that the incidence of ocular hypertension (IOP > 21 mmHg) and cataract formation after single-dose DEX implant were 13.6 (Figure 4A, 95%CI = 3.1, 29.0%, *p* = 0.001, *I*^2^ = 79.7%) and 5.4% (Figure 4B, 95%CI = 0.6, 13.3%, *p* = 0.085, *I*^2^ = 51.1%), respectively. All eyes with intraocular hypertension could be controlled with topical treatments. Two unexpected adverse events including vitreous hemorrhage and lens injury were observed immediately after implantation, which were successfully treated with vitrectomy (Supplementary Table S2). There were no reported cases of endophthalmitis and retinal detachment during the follow-up period. The Egger test demonstrated no significant publication bias of visual and structural outcomes in the present meta-analysis (Supplementary Table S3).

## Discussion

In the present meta-analysis, favorable visual prognosis and significant anatomical improvement were demonstrated after a single-dose DEX implant in patients with refractory UME. No serious adverse events were recorded in the follow-up period. Based on the results, the DEX implant could be an effective therapeutic option for chronic and refractory patients with UME who had previously undergone systemic therapy.

Uveitis occurs in population of all ages but frequently affects working-age individuals, thus posing a substantial socioeconomic burden on the healthcare system (21). Macular edema is the most frequent and sight-threatening complication of non-infectious uveitis, which leads to central visual impairment. Taking the adverse events and side effects of systemic corticosteroid into account, some scholars advocated the intravitreal dexamethasone implant for chronic and refractory UME.

The most noteworthy result emerging from the present meta-analysis was significantly improved BCVA and reduced CMT after the implant of single-dose DEX, indicating the strong ability in inhibiting inflammation and edema during the 6-month follow-up period. Similar findings were also reported in an earlier systematic review, which showed that the DEX implant was an effective option for posterior uveitis and improved the final visual outcome significantly (22). In addition, the therapeutic effects of the DEX implant can maintain for 1 year for macular edema in quiescent uveitis (23). For patients with persistent and chronic UME whose response is unsatisfactory, the DEX implant has the ability to reduce the incidence of visual loss (24). A possible explanation is that dexamethasone can reduce the expression of VEGF, pro-inflammatory cytokines, and chemokines efficiently and then promote the repair of the blood–retinal barrier. It is worthwhile to point out that a small group of patients in included studies underwent repeated injections of the DEX implant due to elevated CMT or deteriorated visual acuity, and most investigations did not provide the relevant results after repeated implants. Longitudinal cohort trials with longer follow-ups are desirable to ensure the reliability and stability of the DEX implant in chronic UME.

Adverse events were relatively rare in the included studies. The most common side effects are ocular hypertension and cataract formation. Previous network meta-analysis including random controlled trials confirmed that the DEX implant had a lower incidence of cataract progressing in non-infectious uveitis (9). Data analyzed from another meta-analysis confirmed that the incidence of increased IOP and cataract were 20.6 and 11%, respectively (22). Thus, it is essential to inform the patients of the potential risks and monitor the lens status and IOP fluctuation after the DEX implant. However, the incidence of cataract formation should be interpreted with caution. It remains unclear whether the cataract was attributed to the DEX implant or related to the natural course of uveitis. In addition, taking the potentially severe complications into consideration, patients with a history of glaucoma and active ocular infection should not be allowed to receive the DEX implant.

Substantial heterogeneity in meta-analyses of this study was identified, which may be due to the various clinical demographics of included participants. The mean disease duration of uveitis or macular edema varied widely and was not reported in some studies, which may contribute to the heterogeneity. Second, the previous systemic and local treatment options varied extensively among studies, which contained oral steroids, immunosuppressants, biological agents, anti-VEGF, etc., also leading to clinical heterogeneity. Another critical concern is the lack of a precise grading method to define and assess the degree of UME, and the significant heterogeneity of statistical analyses is unavoidable. Further investigations, which consider these variables, will need to be conducted.

Several limitations of the present analysis should be considered. First, six studies were designed as retrospective studies and one was a prospective study, and all of the included studies lacked a control group, limiting the reliability of evidence and leading to inevitable inclusion criteria bias. The previous therapeutic strategies were not reported by Nagpal et al., which could also result in selection bias (19). In addition, the relatively small size of the included studies limited the ability to draw definitive conclusions. Moreover, considering the limited data extracted from the included articles, further detailed analysis such as subgroup analysis cannot be performed.

In conclusion, this meta-analysis demonstrated that the DEX implant may play a promising role in the management of patients with persistent and chronic UME. Taking present findings into account, further investigations featuring multicenter and random control should be performed to evaluate the long-term effect and potential complications of repeated injections of DEX.

## Data availability statement

The original contributions presented in the study are included in the article/Supplementary material, further inquiries can be directed to the corresponding authors.

## Author contributions

SF and X-yS designed the review and extracted the data. C-fZ, ZC, JY, and S-pY contributed to the analysis of data. SF drafted the article. JL and XL reviewed and edited the manuscript. All authors contributed to the article and approved the submitted version.

## Funding

This study was supported by the Lishui Municipal Science and Technology Project (2023GYX66) and the Youth Fund Program of Lishui Municipal Central Hospital (2022qnjj15).

## Conflict of interest

The authors declare that the research was conducted in the absence of any commercial or financial relationships that could be construed as a potential conflict of interest.

## Publisher’s note

All claims expressed in this article are solely those of the authors and do not necessarily represent those of their affiliated organizations, or those of the publisher, the editors and the reviewers. Any product that may be evaluated in this article, or claim that may be made by its manufacturer, is not guaranteed or endorsed by the publisher.
