# Supplementary material for: Efficacy and safety of single-dose intravitreal dexamethasone implant in non-infectious uveitic macular edema: A systematic review and meta-analysis
Source: Front Med (Lausanne). 2023 Feb 17;10:1126724. doi: 10.3389/fmed.2023.1126724 (PMC9982842; doi:10.3389/fmed.2023.1126724)
Supplement: Supplementary file 1 [file Table_1.DOCX]

Supplementary Material

**Table S1.** Clinical demographics of included studies

| Study | Clinical diagnoses (patients/percentage) | | | | | | | Baseline IOP (mmHg) | Phakic/Pseudophakic (eyes) |
| --- | --- | --- | --- | --- | --- | --- | --- | --- | --- |
|  | BD | VKH | IU | Idiopathic | Sarcoidosis | BSRC | Other |  |  |
| Bansal et al | 2(7.4%) | No | 6(22.2%) | 8(29.6%) | 2(7.4%) | No | 9(33.3%) | NA | NA |
| Garweg et al | NA | | | | | | | 13.0±1.0 | NA |
| Fabiani et al | 4(18.2%) | 2(9.1%) | No | 16(72.7%) | No | No | No | NA | 14/8 |
| Cardoso et al | 2(6.5%) | 2(6.5%) | No | 15(48.4%) | 9(29%) | 1(3.2%) | 2(6.5%) | NA | NA |
| Tsang et al | No | No | No | 7(46.7%) | 3(20%) | 2(13.3%) | 3(20%) | NA | NA |
| Nagpal et al | NA | | | | | | | 18.2±4.1 | 23/7 |
| Yalcinbayir et al | 20(100%) | No | No | No | No | No | No | 13.1±2.4 | 23/4 |

BD: Behcet disease; VKH: Vogt-Koyanagi-Harada; IU: Intermediate uveitis; BSRC: Birdshot retinochoroidopathy; IOP: Intraocular pressure;

NA: Not applicable.

**Table S2.** Detailed demographics of included studies

| Study | Other complications (eyes) | | | | Eyes with ocular hypertension | |
| --- | --- | --- | --- | --- | --- | --- |
|  | RD | Lens injury | Ocular infection | VH | Pseudophakic % | Range of IOP (mmHg) |
| Bansal et al | No | No | No | No | NA | NA |
| Garweg et al | NA | NA | NA | NA | NA | NA |
| Fabiani et al | No | No | No | No | NA | 23-28 |
| Cardoso et al | No | No | No | 1 | NA | NA |
| Tsang et al | No | 1 | No | No | NA | NA |
| Nagpal et al | No | No | No | No | NA | NA |
| Yalcinbayir et al | No | No | No | No | NA | NA |

RD: Retinal detachment; VH: Vitreous hemorrhage; IOP: Intraocular pressure; NA: Not applicable.

**Table S3** Publication bias of BCVA and CMT during follow-up period. (Number of included studies≥4)

| Outcome variables | Number of studies | Egger test (p value) |
| --- | --- | --- |
| BCVA |  |  |
| 1month | 5 | 0.570 |
| 3 months | 6 | 0.828 |
| 6 months | 4 | 0.635 |
| CMT |  |  |
| 1 month | 7 | 0.982 |
| 3 months | 7 | 0.929 |
| 6 months | 5 | 0.124 |
